# Anemia in the Elderly: not Always what it Seems

**DOI:** 10.4084/MJHID.2016.017

**Published:** 2016-02-25

**Authors:** Marco Cerrano, Elena Crisà, Valentina Giai, Mario Boccadoro, Dario Ferrero

**Affiliations:** 1 Hematology Division, Università degli Studi di Torino, Turin, Italy

## Abstract

Anemia in the elderly is a common but challenging clinical scenario. Here we describe the case of an older woman who presented with anemia and elevated inflammation markers. After a complete diagnostic work-up, a definite etiology of the anemia could not be found so eventually a bone marrow biopsy was performed and she was diagnosed with myelodysplastic syndrome. She responded well to erythropoietin treatment but her inflammation markers remained elevated thus a positron emission tomography was performed. It turned out that the patient suffered from giant cell artheritis and her anemia completely resolved after steroid treatment. Our case outlines that it is necessary to pay particular attention to anemia of inflammation, which could be due to several and often masked conditions. Myelodysplatic syndromes should be considered when other causes have been ruled out, but their diagnosis can be difficult and requires expertise in the field.

## Introduction

Anemia in the elderly is a very common condition that contributes to morbidity and mortality and significantly impairs quality of life. In the Third National Health and Nutrition Examination Survey (NHANES III) study the incidence of anemia in men and women older than 65 was 11% and 10.2%, respectively,^1^ thus representing a problem almost every physician deals with. Several causes often contribute to anemia in this age group, and it is not always possible to find a unifying diagnosis,^2^ hence it frequently represents a challenging clinical scenario. Here we present the case of an elderly Italian woman investigated for persistent anemia and elevated inflammation markers.

## Case Report

### Case presentation

A 68 year old woman was admitted to a primary care center for worsening asthenia and fatigability. During the two months before admission, she also referred weight loss, mild fever, and cough.

### Clinical history

She was diagnosed with ovarian cancer 13 years before, and she was treated with surgery and a carboplatin-containing chemotherapy regimen. No signs of disease recurrence were found afterwards. She suffered from hypertension, well controlled with medical therapy, arthrosis, and chronic constipation.

### Initial work-up

At admission clinical examination was unremarkable. Blood count showed severe normocytic anemia (hemoglobin 7.5g/dL, mean corpuscular volume 90 fL) and mild thrombocytosis (platelets 452000/μL) while reticulocyte count, leucocyte count, and differential were normal. Iron or vitamin deficiency were ruled out, and lactate dehydrogenase, serum creatinine, thyroid functioning and liver tests were normal. Reactive C protein (RCP), erythrocyte sedimentation rate (ESR) and serum ferritin, however, were significantly increased (18.2 mg/dL, 160 mm/h and 1136 ng/mL, respectively).

### Differential diagnosis

Nutritional deficiencies (i.e. lack of iron, vitamin B12 or folic acid), chronic kidney disease, thalassemia trait and inflammation are the most frequent causes of anemia in elderly patients and should be considered first.^3^ Hemolysis, radio- and chemo-therapeutic interventions, hypothyroidism or hepatic insufficiency are other common causes that should be ruled out. Anemia due to inflammation (AI) is maybe the most complex one. As a matter of fact, several pathophysiological mechanisms are involved such as disturbance of iron homeostasis, impaired proliferation of erythroid progenitor cells and reduced erythropoietin response. Furthermore, the causes of inflammation are often multiple and not always the source is apparent (e.g. inflammatory bowel disease, rheumatologic disorders, cancer, infections).^4^

Besides, AI can mask iron deficiency because, in the presence of both these conditions, the commonly used serum iron-status indicators (namely iron, transferrin, transferrin saturation and ferritin) can be difficult to interpret.^3^ The discovery of hepcidin, the main regulator of iron homeostasis, significantly improved our understanding of the pathophysiology of AI and the measurement of serum hepcidin level, which is down-regulated in case of iron deficiency and up-regulated in presence of inflammation, soon could become a useful tool in the diagnostic work-up of anemia in the elderly.^5^

### Further examinations

Since inflammation markers remained elevated in our patient, other investigations were performed to rule out an infection, an autoimmune disease or malignancy: microbiological tests were negative, autoimmunity tests showed a low antinuclear antibody titer and a weak rheumatoid factor positivity, chest, and abdomen computed tomography and echocardiography were normal. Anemia persisted, no precise inflammatory source could be found and a bone marrow (BM) trephine biopsy was eventually performed and evaluated by the local pathologist. It showed a hypercelullar bone marrow with trilinear dysplasia, abnormal localization of immature precursors and 1.5% of blasts. She was diagnosed with myelodysplastic syndrome (MDS)-unspecified, without blast excess, likely secondary to chemotherapy exposure. Cytogenetic analysis and BM aspiration were not performed.

### Differential diagnosis

A BM dysfunction, either primary, such as MDS, or secondary to neoplastic or infectious agents, should be considered after ruling out all other causes of anemia. MDS are suspected in elderly patients in case of unexplained cytopenia, more commonly isolated macrocytic anemia, and confirmed with a BM aspirate and biopsy.

### Hematologic follow-up

The patient was then discharged and referred to our center. When she presented to our clinic, she was afebrile, reported fatigue and weakness. Her blood count was stable with isolated anemia requiring weekly transfusions and ESR and RCP remained elevated. In order to confirm the diagnosis, we proposed her a new BM evaluation with morphological and cytogenetic analysis but the patient refused it. As per our policy, we tested the blood level of Wilms tumor gene transcript (frequently elevated in acute myeloid leukemia and MDS) 6, and it was in the normal range. Giving the diagnosis of low risk MDS with isolated anemia, we checked serum erythropoietin level, that resulted in the reference range but inadequate for the degree of anemia (31.7 mUI/mL), therefore we started the patient on recombinant erythropoietin, 40000 U weekly. Her anemia progressively improved and she achieved an erythroid response^7^ after 3 weeks of treatment, becoming transfusion independent.

### Final diagnosis

**Figure 1:**
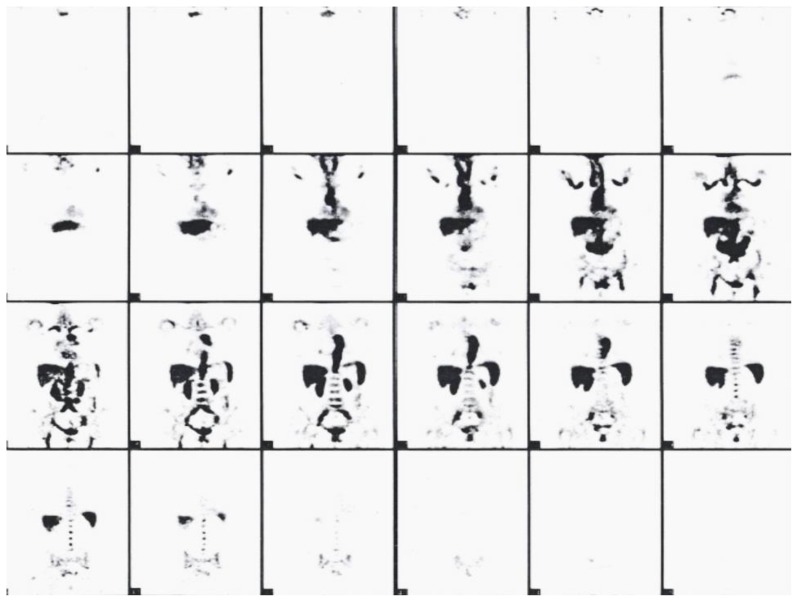
The total body positron emission tomography (PET) revealed a dishomogeneous and intense hyperfixation in the wall of medium and big arterial vessels.

So far it could have looked like a typical low-risk MDS responsive to erythropoietin. However, the patient was still complaining of arthralgias and ESR and RCP remained elevated. Moreover, she reported the appearance of a livedo reticularis on her lower limbs. To better clarify the case, given the persistently elevated inflammation markers and the personal history of ovarian cancer, a total body positron emission tomography (PET) was performed. Surprisingly, it revealed a dishomogeneous and intense hyperfixation in the wall of medium and big arterial vessels, consistent with a vascular inflammatory process (Figure 1). We referred the patient to a rheumatologist, and she underwent a biopsy of the temporal artery which revealed histology consistent with giant cell arteritis (GCA). Erythropoietin was discontinued; she was started on corticosteroid therapy and her anemia rapidly resolved. Seven years after GCA diagnosis our patient is doing well, with normal blood counts and without any further therapy.

## Discussion

In the case we are presenting, the initial work-up of our patient demonstrated severe anemia associated with significantly elevated inflammation markers, prompting the diagnosis of AI. However, even though several examinations were performed, a clear etiology could not be found and eventually the patient underwent a BM biopsy. The diagnosis seemed to be MDS.

MDS usually present with anemia, isolated or combined with neutropenia and thrombocytopenia,^8^ but anemia and thrombocytosis can seldom occur.^9^,^10^ Anemia is usually macrocytic (rarely it can be normocytic or even microcytic),^11^ reticulocyte count is (relatively) reduced, there can be signs of dyserithropoiesis and serum ferritin level can be elevated. On peripheral blood smear a dimorphic red-cell population that includes oval macrocytes can be seen, often together with neutrophils or platelets abnormalities (e.g. hypogranulated neutrophils or pseudo Pelger–Huët cells).^8^ MDS diagnosis is confirmed by performing a BM examination. It is important to carry out both BM aspirate and trephine biopsy as the first one is essential to evaluate cellular morphology and to count the proportion of blasts while the latter allows for determination of BM cellularity and architecture.^12^ Indeed the pathological hallmark of MDS is BM dysplasia that can only be assessed by morphology, which represents the most important tool to establish a definite diagnosis.^13^ The 2008 World Health Organization classification of MDS requires the demonstration of unequivocal dysplasia in at least 10% of the cells of the erythroid, granulocytic or megakaryocytic lineage.^14^ However, nutritional deficiencies, medications, toxins, growth factor therapy, inflammation or infections can sometimes cause secondary dysplasia and thus they should be excluded before a diagnosis of MDS is established. In the cases of MDS with an excess of blasts or with more than 15% of ring sideroblasts in the erythroid precursors the diagnosis is usually straightforward. In the other cases, the morphologic evidence of dysplasia may not be unequivocal, and the presence of a specific cytogenetic abnormality or the immunophenotyping can be helpful in confirming the diagnosis.^14^,^15^ If unilinear dysplasia is the only proven sign of myelodysplasia an observation period of 6 months and a second BM investigation is recommended to establish a definite MDS diagnosis.^14^ The complexity of diagnosis of MDS can lead to mistakes and significant discrepancies between peripheral and tertiary care centers have been outlined.^16^,^17^ Therefore, it appears important that an experienced hematologist along with an hemopatologist establish the diagnosis of MDS and a discussion with other colleagues experts in this field could be useful when in doubt.

Our patient was eventually diagnosed with GCA. GCA can present with typical symptoms such as a headache, jaw and tongue claudication, scalp tenderness, visual disturbance and manifestations of polymyalgia rheumatica^18^ and the diagnosis still relies on the 1990 American College of Rheumatology classification criteria [3 of the following 5 criteria are required: 1) age 50 years or older, 2) new-onset localized headache, 3) temporal artery tenderness or decreased temporal artery pulse, 4) ESR of at least 50 mm/h, 5) abnormal artery biopsy specimen characterized by mononuclear infiltration or granulomatous inflammation].^19^ However, the diagnosis of GCA can be difficult due to the variability of clinical presentation, and the utility of these criteria in clinical practice has been questioned.^20^ Furthermore, the presentation of GCA can be atypical and cases showing only anemia and raised inflammatory markers have been reported.^21^ A persistent dry cough can be a presenting symptom too, either isolated or associated with typical manifestations.^22^ Our case was challenging because the patient complained only of anemia related symptoms, arthralgias, and mild fever and the elevated inflammation markers could not be easily interpreted. It outlines that is it is necessary to be aware of the entire spectrum of symptoms of GCA in order to consider it in the differential diagnosis of anemia. Indeed, our patient initially complained of a cough, but this symptom was not reported later. The use of PET can certainly facilitate the diagnosis of GCA, but it is expensive, and it exposes patients to a significant radiation risk.

An association between autoimmune diseases, including vasculitis, and MDS has been reported, especially in case of chronic myelo-monocytic leukemia and high risk MDS.^23^–^25^ However, in our patient anemia completely resolved with steroid treatment and she is currently doing well seven years after GCA diagnosis without any further therapy.

## Conclusion

Our case reminds clinicians they should always consider multiple differential diagnosis of anemia in the presence of systemic inflammation, which can be itself a cause of secondary dysplasia. MDS can be considered when other etiologies are excluded, but it should be kept in mind that the diagnosis is difficult, especially when only anemia is present, and it is mostly based on morphology and histopathology expertise.
